# Supplementary figures and images for: Rootstock Selection for Resisting Cucumber Fusarium Wilt in Hainan and Corresponding Transcriptome and Metabolome Analysis
Source: Plants (Basel). 2025 Jan 24;14(3):359. doi: 10.3390/plants14030359 (PMC11820677; doi:10.3390/plants14030359)

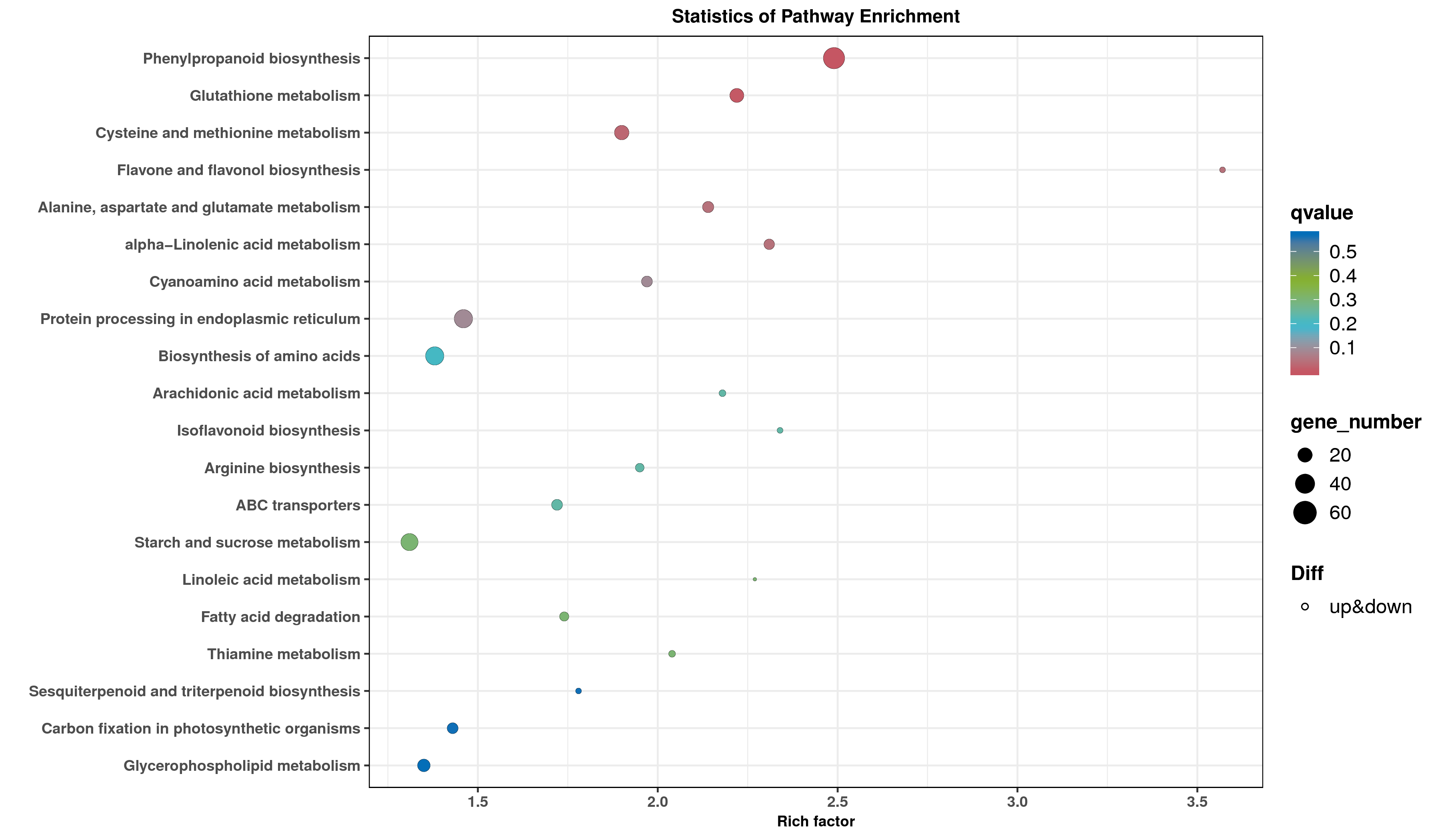

Supplement: Supplementary file 1 [file plants-14-00359-s001.zip › Non-published Material/FigureS1.png]

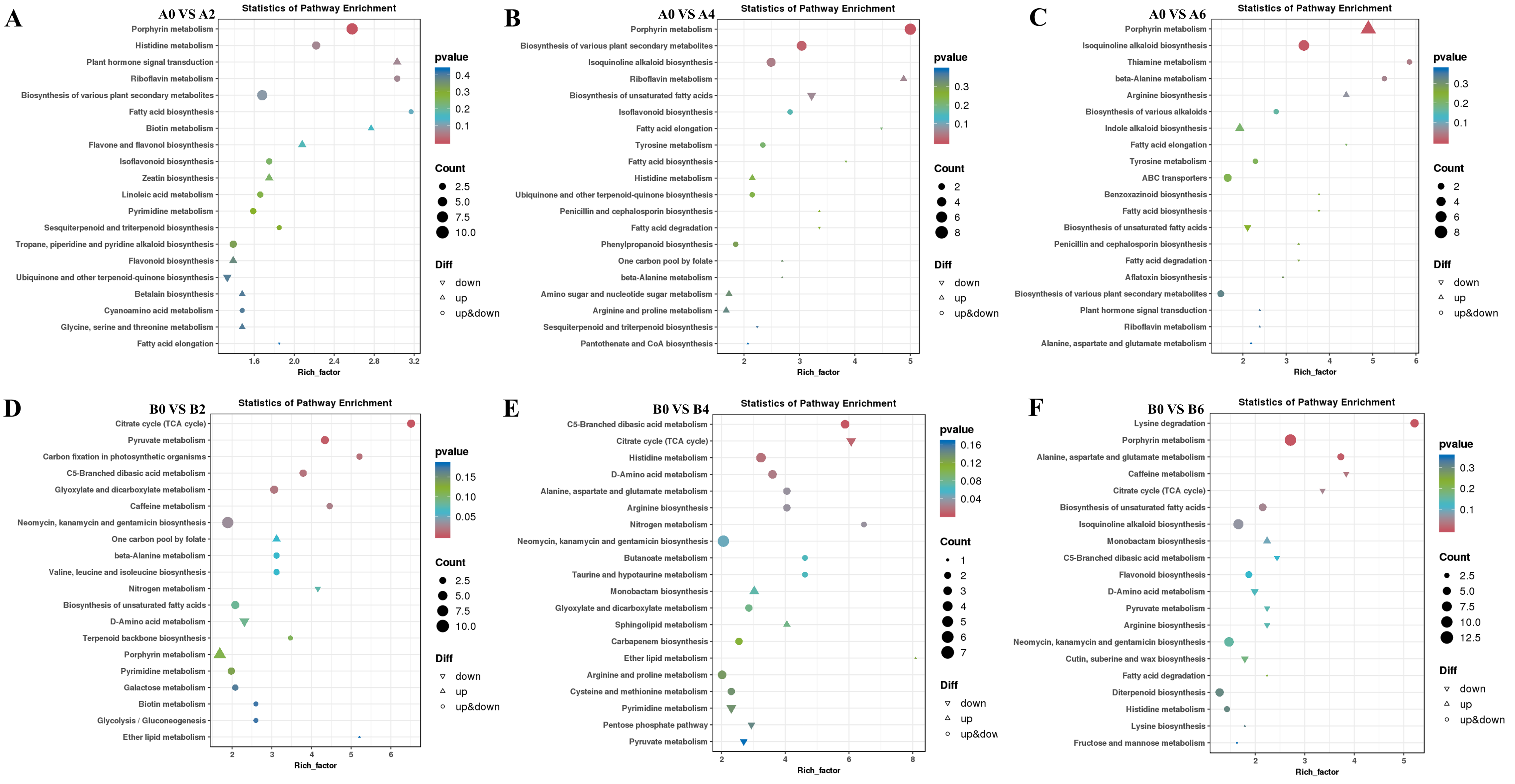

Supplement: Supplementary file 1 [file plants-14-00359-s001.zip › Non-published Material/FigureS2.tif]

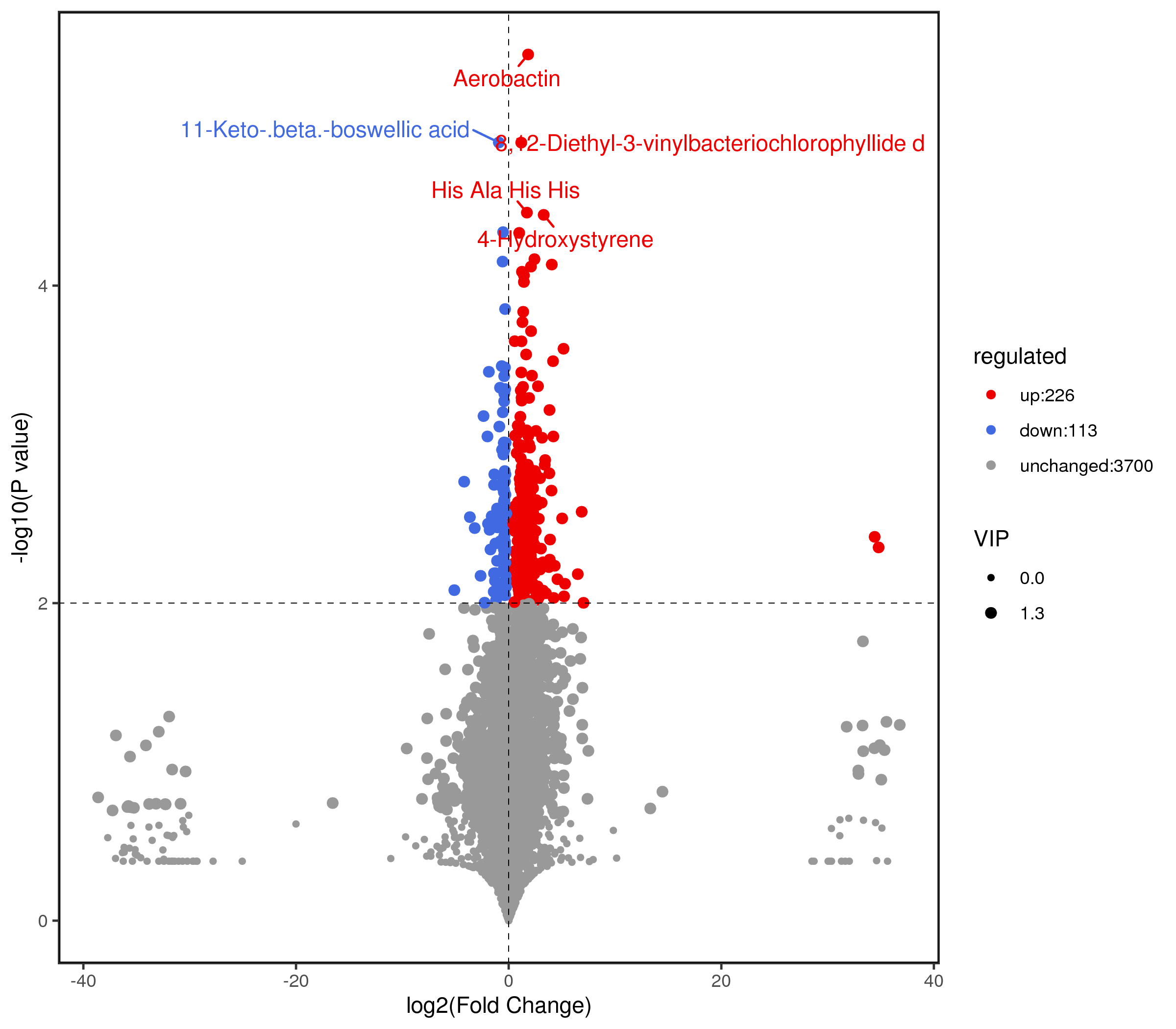

Supplement: Supplementary file 1 [file plants-14-00359-s001.zip › Non-published Material/FigureS3.png]

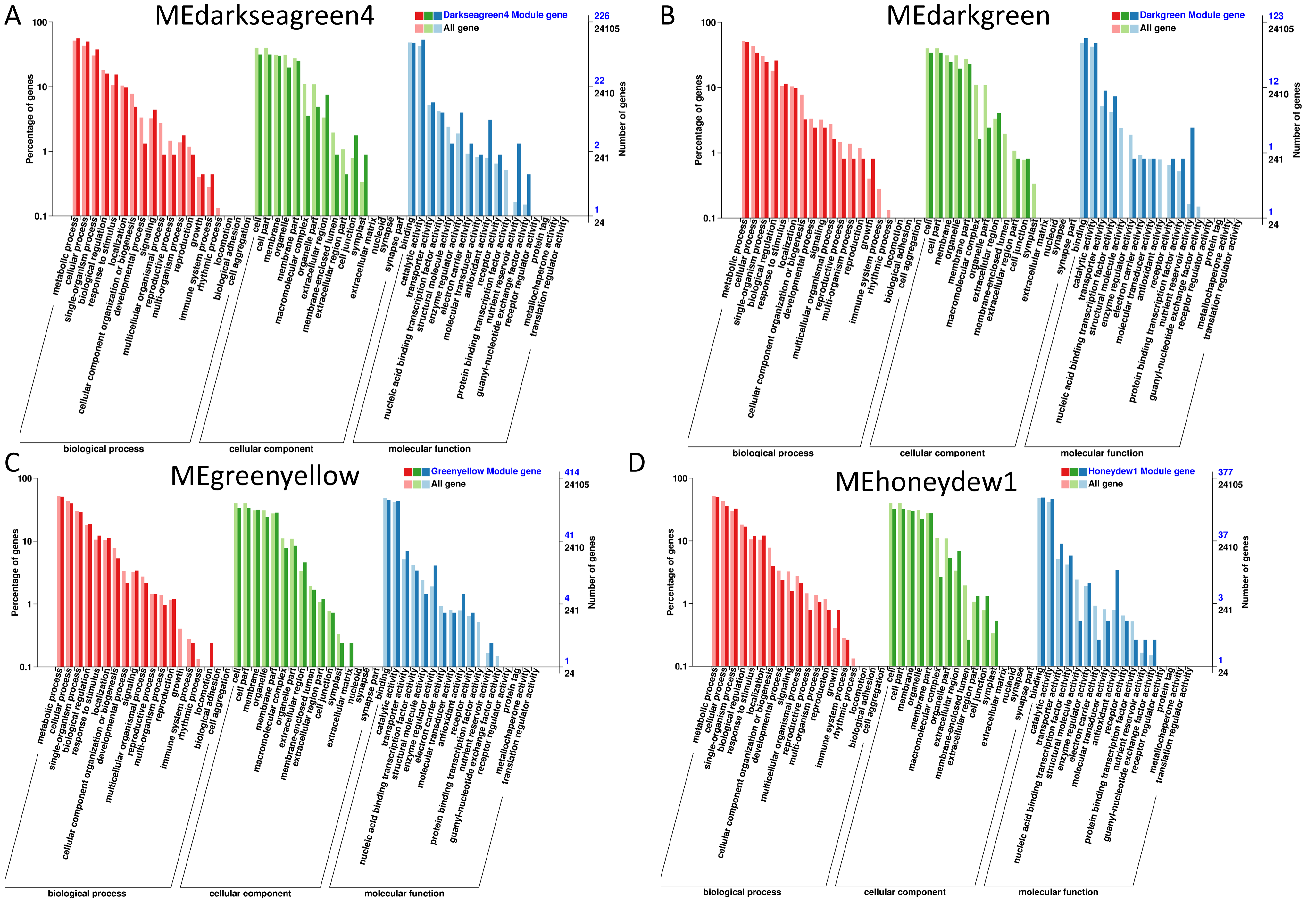

Supplement: Supplementary file 1 [file plants-14-00359-s001.zip › Non-published Material/FigureS4.tif]

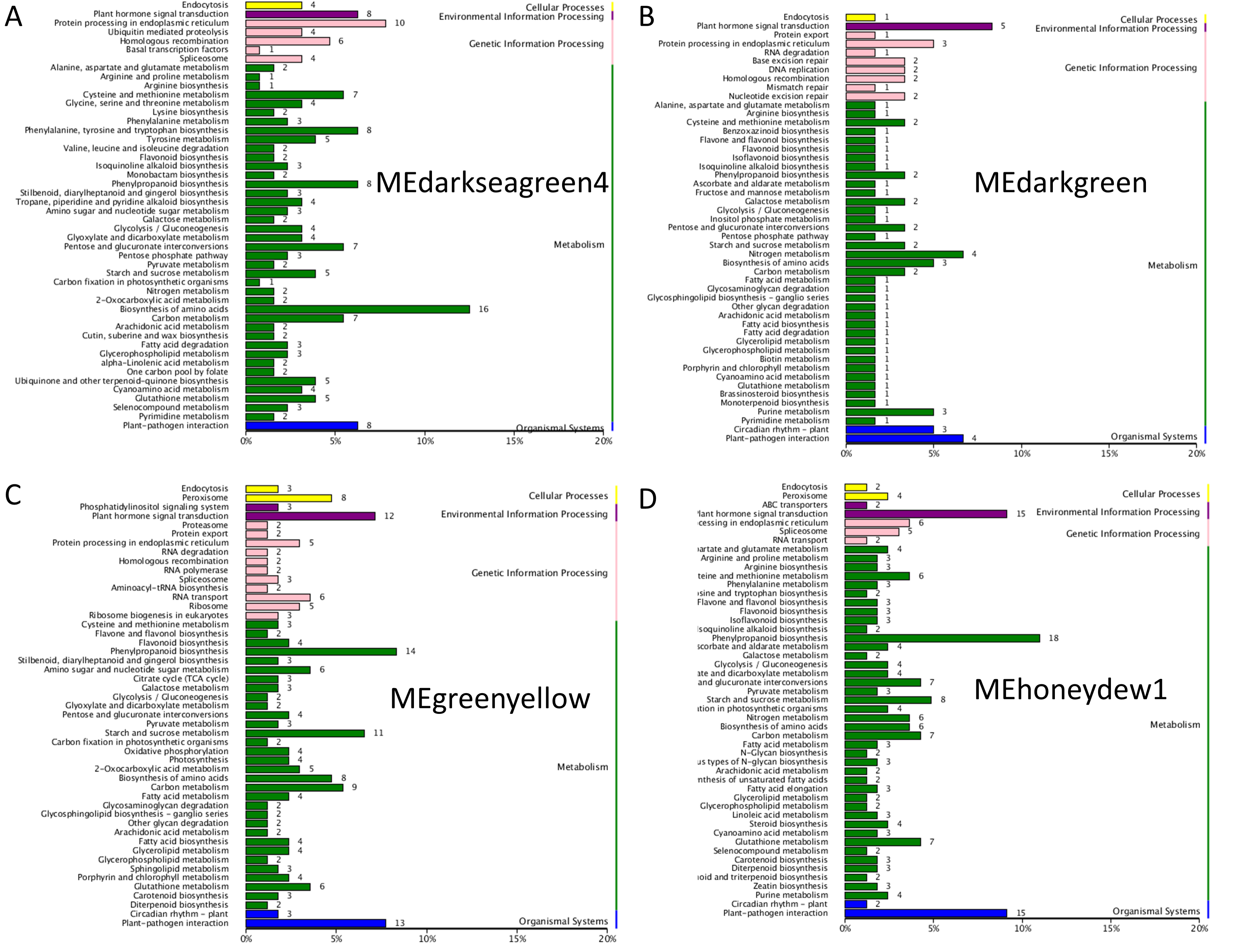

Supplement: Supplementary file 1 [file plants-14-00359-s001.zip › Non-published Material/FigureS5.tif]
